# Autobiographical memory and default mode network function in schizophrenia: an fMRI study

**DOI:** 10.1017/S0033291719003052

**Published:** 2021-01

**Authors:** Marta Martin-Subero, Paola Fuentes-Claramonte, Pilar Salgado-Pineda, Josep Salavert, Antoni Arevalo, Clara Bosque, Carmen Sarri, Amalia Guerrero-Pedraza, Aniol Santo-Angles, Antoni Capdevila, Salvador Sarró, Raymond Salvador, Peter J. McKenna, Edith Pomarol-Clotet

**Affiliations:** 1 FIDMAG Germanes Hospitalàries Research Foundation, Barcelona, Spain; 2 CIBERSAM (Centro de Investigación Biomédica en Red de Salud Mental), Madrid, Spain; 3 Department of Psychiatry and Forensic Medicine, Universitat Autònoma de Barcelona, Barcelona, Spain; 4 Psychiatry Department, Hospital Sant Rafael, Barcelona, Spain; 5 Psychiatry Department, Hospital Sagrat Cor Martorell Barcelona, Barcelona, Spain; 6 Benito Menni Centre Assistencial en Salut Mental, Sant Boi de Llobregat, Barcelona, Spain; 7 Radiology Unit, Hospital de la Santa Creu i Sant Pau (HSCSP), Barcelona, Spain; 8 CIBER-BBN (Centro de Investigación Biomédica en Red en Bioingeniería, Biomateriales y Nanomedicina), Zaragoza, Spain

**Keywords:** Autobiographical memory, default mode network, fMRI, schizophrenia

## Abstract

**Background:**

The brain functional correlates of autobiographical recall are well established, but have been little studied in schizophrenia. Additionally, autobiographical memory is one of a small number of cognitive tasks that activates rather than de-activates the default mode network, which has been found to be dysfunctional in this disorder.

**Methods:**

Twenty-seven schizophrenic patients and 30 healthy controls underwent functional magnetic resonance imaging while viewing cue words that evoked autobiographical memories. Control conditions included both non-memory-evoking cues and a low level baseline (cross fixation).

**Results:**

Compared to both non-memory evoking cues and low level baseline, autobiographical recall was associated with activation in default mode network regions in the controls including the medial frontal cortex, the posterior cingulate cortex and the hippocampus, as well as other areas. Clusters of de-activation were seen outside the default mode network. There were no activation differences between the schizophrenic patients and the controls, but the patients showed clusters of failure of de-activation in non-default mode network regions.

**Conclusions:**

According to this study, patients with schizophrenia show intact activation of the default mode network and other regions associated with recall of autobiographical memories. The finding of failure of de-activation outside the network suggests that schizophrenia may be associated with a general difficulty in de-activation rather than dysfunction of the default mode network *per se*.

Autobiographical recall refers to the conscious re-imagining of events from one's past, with the memories typically being accompanied by some of their original sensory and emotional qualities (Rubin, 1996; Svoboda *et al*., 2006). Autobiographical memory forms part of the broader construct of episodic memory, but differently from standard episodic memory tasks, which employ experimenter-generated stimuli such as lists of words, it is tested by asking subjects to recall memorable events in their own lives. These may be elicited in response to cue words like ‘river’ or ‘puppy’ (the Crovitz task; Crovitz and Schiffman, 1974), or by means of prompts about events such as starting a new job or attending a wedding (the Autobiographical Memory Test, AMI; Kopelman *et al*., 1989). Autobiographical memory has been linked conceptually to the ability to imagine future events, and together they form the concept of ‘mental time travel’ (Schacter *et al*., 2007). Autobiographical memory has also been argued to play a key role in the construction of one's sense of self (Conway and Pleydell-Pearce, 2000).

As expected, given the evidence for episodic memory impairment in the disorder (e.g. Palmer *et al*., 2009), autobiographical memory has been found to be impaired in schizophrenia. A meta-analysis of 20 studies (Berna *et al*., 2016) found significantly poorer performance compared to healthy controls in all aspects of autobiographical recall examined; effect sizes were large for richness of detail and specificity of memories, and moderate for conscious recollection, i.e. the degree of personal awareness of participating in the re-experienced events.

To date, there has been only one study of the brain functional correlates of autobiographical recall in schizophrenia: Cuervo-Lombard *et al*. (2012) compared 13 schizophrenic patients and 14 healthy controls using a task where they saw cue words and pressed a button when they recalled a personal event associated with them. The control task consisted of a button press in response to instructions to use either the middle or index finger to do this. Whole-brain functional magnetic resonance imaging (fMRI) with correction for multiple comparisons revealed no major clusters of significant difference between the groups in the cortex, but there were small clusters of reduced activation in the patients in the lateral ventral tegmental area, the right cerebellum and both caudate nuclei. Uncorrected comparisons within a mask comprising the areas activated by the patients and/or the controls, however, revealed additional areas of reduced activation in the patients in the medial frontal cortex, the precuneus, the left lateral prefrontal cortex, the left medial temporal lobe and the occipital cortex.

Autobiographical recall is also of interest from the functional imaging point of view because it has been found to activate the so-called default mode network (Buckner *et al*., 2008; Raichle, 2015). This network consists of a set of brain regions that are normally active at rest but which de-activate during performance of a wide range of attention-demanding tasks. It includes prominently two midline areas, the medial prefrontal cortex and the posterior cingulate cortex/precuneus, as well as parts of the parietal and temporal lobe cortex and the hippocampus (Gusnard and Raichle, 2001; Raichle *et al*., 2001; Buckner *et al*., 2008). The small number of tasks that have been found to activate rather than de-activate the default mode network regions include imagining the future (Schacter *et al*., 2007), making judgements about oneself and others (van der Meer *et al*., 2010; Murray *et al*., 2012), making moral judgements (Boccia *et al*., 2017), engaging in theory of mind-type reasoning (Schurz *et al*., 2014) and autobiographical memory. With respect to this last paradigm, Svoboda *et al*. (2006) meta-analysed 24 positron-emission tomography (PET) and fMRI studies using autobiographical memory tasks and found pooled evidence of activations in the medial frontal cortex and the retrosplenial/posterior cingulate cortex, i.e. the two midline cortical ‘nodes’ of the default mode network, as well as other regions including the dorsolateral prefrontal cortex (DLPFC), the ventrolateral prefrontal cortex, other lateral prefrontal regions, the medial and lateral temporal cortex, the temporoparietal junction and the cerebellum.

Default mode network dysfunction during performance of various cognitive tasks has been reported in schizophrenia since 2007. Two initial studies (Garrity *et al*., 2007; Harrison *et al*., 2007) found increased de-activation or a mixed pattern of increased activation and failure of de-activation, respectively. Since then, however, the almost invariable finding has been failure of de-activation, which is typically seen in the medial frontal cortex (Pomarol-Clotet *et al*., 2008; Whitfield-Gabrieli *et al*., 2009; Mannell *et al*., 2010; Salgado-Pineda *et al*., 2011; Schneider *et al*., 2011; Dreher *et al*., 2012; Haatveit *et al*., 2016), although the posterior cingulate gyrus/precuneus has also sometimes been found to be affected (Salgado-Pineda *et al*., 2011; Schneider *et al*., 2011). There appear to be only two exceptions: using a visual working memory task with various levels of difficulty, Hahn *et al*. (2017) found that 21 schizophrenic patients and 16 controls showed no differences in de-activation across 13 regions of interest placed in the default mode network, and at the two hardest levels de-activation was significantly greater in the patients. In another study using a task requiring direction of attention to visual stimuli that either predicted or did not predict the location of a subsequent target, the same group (Hahn *et al*., 2016) again found no differences in default mode network de-activation in 20 schizophrenic patients compared to 20 healthy controls (when the cue was predictive) or greater de-activation (when the cue was non-predictive).

Given the evidence for failure of default mode de-activation (and perhaps increased de-activation in some circumstances) in schizophrenia, how the network behaves during a task like autobiographical memory, which normally activates it, is clearly of some interest. In the current study, we examined both activations and de-activations associated with autobiographical recall in schizophrenia, using a larger sample of patients and controls than in Cuervo-Lombard *et al*.'s (2012) study and employing whole-brain analysis with correction for multiple comparisons.

## Methods

### Subjects

The patient sample consisted of 27 right-handed patients meeting DSM-IV criteria for schizophrenia, recruited from three psychiatric hospitals in Barcelona (Benito Menni CASM, Hospital Sagrat Cor de Martorell and Sant Rafael Hospital). The diagnosis was established using the Structured Clinical Interview for DSM Disorders (SCID) (First *et al*., 2002). Patients were excluded if they (a) were younger than 18 or older than 65, (b) had a history of brain trauma or neurological disease or (c) had shown alcohol/substance abuse/dependence within 12 months prior to participation. With respect to the last criterion, all participants were questioned about alcohol and drug use during the previous year, and we also excluded those who reported habitual use of cannabis. Social use of alcohol was permitted, as was non-habitual use of cannabis. All the patients were taking antipsychotic treatment (23 on atypical neuroleptics, one on typical neuroleptics and three on both).

The control sample consisted of 30 right-handed healthy individuals recruited from non-clinical staff working in the hospitals, their relatives and acquaintances, plus independent sources in the community. They met the same exclusion criteria as the patients and they were also interviewed using the SCID to exclude current and past psychiatric disorders. They were questioned and also excluded if they reported a history of treatment with psychotropic medication beyond non habitual use of night sedation.

The two groups were selected to be matched for age, sex and estimated IQ (premorbid IQ in the patients). This latter was measured using the Word Accentuation Test (Test de Acentuación de Palabras, TAP; Del Ser *et al*., 1997; Gomar *et al*., 2011). All patients were scanned when in a relatively stable condition.

All participants gave written informed consent. All the study procedures were approved by the local research ethics committee.

### Autobiographical memory task

The task used was based on the one developed by Oertel-Knochel *et al*. (2012), which employed personalised cues that had previously been found to evoke autobiographical memories in the subjects. While they employed a control which involved completing a sentence with a semantically appropriate word, we changed this to one involving viewing of cues that had previously been found not to evoke autobiographical memories.

Before the fMRI session, each participant was administered the cue words from the Crovitz test (Crovitz and Schiffman, 1974) and the autobiographical prompt phrases from the AMI (Kopelman *et al*., 1989), in order to generate between four and six autobiographical memories for each of the time periods covering childhood, adolescence, adulthood and the preceding year. The stimuli chosen for the fMRI paradigm consisted of groups of three words personalised for each participant. The first word in the group referred to one of the above four time periods, and the other two words were chosen on the basis that they had previously evoked autobiographical memories (e.g. *childhood-grandmother-cake*; *adult-car-robbery*). All memories were required to have been given the maximum score of 3 in the AMI, indicating that they were clearly specified in time and place and descriptively rich. For the control conditions, we randomly selected groups of three words from the words that did not evoke autobiographical memories.

Ten blocks of non-memory-evoking stimuli were alternated with ten blocks of memory-evoking stimuli; all blocks lasted 20 s. Each block contained two cue sentences of the appropriate type. Subjects were instructed to recollect the memory previously evoked by the three word phrase, or in the case of the non-memory-evoking phrase, to read the phrase with no further requirements. A low level baseline condition was also employed, cross fixation. This was presented between blocks for 16 s.

At the end of the scanning session all participants were asked what they had been thinking about during each condition. Specifically, we asked if they could recollect the memories that they reported in the previous interview during the memory-evoking condition, and whether they were wide awake and focused during the session. Participants who responded negatively to either of these questions were excluded.

### Image acquisition

Images were acquired with a 3T Philips Achieva scanner (Philips Medical Systems, Best, The Netherlands). Functional data were acquired using a T2*-weighted echo-planar imaging (EPI) sequence with the following acquisition parameters: TR = 2000 ms, TE = 30 ms, flip angle = 78°, in-plane resolution = 3 × 3 mm, FOV = 240 mm, slice thickness = 3 mm, inter-slice gap = 1 mm. The autobiographical memory task consisted of 360 volumes. Slices (32 per volume) were acquired with an interleaved order parallel to the AC-PC plane. Before the functional sequences, a high-resolution anatomical 3D volume was acquired using a Turbo Field Echo sequence for anatomical reference and inspection (TR = 8.15 ms; TE = 3.73 ms; flip angle = 8°; voxel size = 0.9375 × 0.9375 mm; slice thickness = 1 mm; slice number = 160; FOV = 240 mm).

Any subjects with excessive head movement during the fMRI sequence, defined as an estimated maximum absolute movement >3.0 mm or an average absolute movement >0.3 mm, were excluded.

### Image preprocessing and analysis

Preprocessing and analysis was carried out with the FEAT module included in the FSL (FMRIB Software Library) software (Smith *et al*., 2004). The first 20 s, corresponding to signal stabilisation, were discarded. Preprocessing included motion correction (using the MCFLIRT algorithm) and co-registration and normalisation to a common stereotactic space (MNI template). Before group analyses, normalised images were spatially filtered with a Gaussian filter (FWHM = 5 mm).

Statistical analysis was performed by means of a general linear model (GLM). Two regressors of interest were defined at the single-subject level analysis (memory-evoking blocks and non-memory-evoking blocks) and the GLM was fitted to generate activation maps of each condition compared to baseline and the comparison between conditions. Group comparisons between patients and controls were performed within the FEAT module, with mixed-effects GLMs (Beckmann *et al*., 2006).

Statistical tests were carried out at the cluster level with a corrected *p* value of 0.05 using Gaussian random field methods. A threshold of *z* = 3.1 was used to define the initial set of clusters.

### Contrasts used in the analysis

In order to examine autobiographical memory associated activations, the main contrast employed was that between cues that evoked and did not evoke autobiographical memories, something that should eliminate ‘noise’ due to aspects of performance common to both tasks.

For de-activations, we focused on the contrast between memory-evoking cues and the low level baseline. This was to avoid a methodological problem identified by Gusnard and Raichle (2001), that examination of relative changes between two active tasks will not necessarily reveal the true picture of activations and de-activations. Specifically, because fMRI analysis is subtractive, task-associated decreases in activation will be obtained not only when there is greater *de-activation* from baseline levels in the task of interest (in this case memory evoking cues) than in the control task (in this case non-memory-evoking cues), but also if there is greater *activation* from baseline levels in the control task than in the task of interest. It follows that de-activations can only be confidently identified with respect to a low level baseline.

## Results

### Demographic data

Age, sex and TAP-estimated IQ data for the patients and controls are shown in Table 1. As can be seen, the two groups were matched on all three variables. None of the patients and two of the controls reported sporadic use of cannabis.

**Table 1.** Demographic data for the patients and controls

	Healthy controls (*N* = 30)	Schizophrenia patients (*N* = 27)	Differences
Sex (M/F)	19/11	17/10	*p* = 0.98
Age	40.40 (11.23)	40.15 (8.75)	*p* = 0.93
Premorbid IQ	103.70 (6.38)	100.23 (10.90)	*p* = 0.16
No. reporting social alcohol use	14	7	
No. reporting non-habitual cannabis use	2	0	
Antipsychotic dose (chlorpromazine equivalents)	–	495.41 (459.01)	

Values for age and premorbid IQ are means (sd)

### fMRI findings: memory-evoking *v.* non-memory evoking cues

**Fig. 1.**
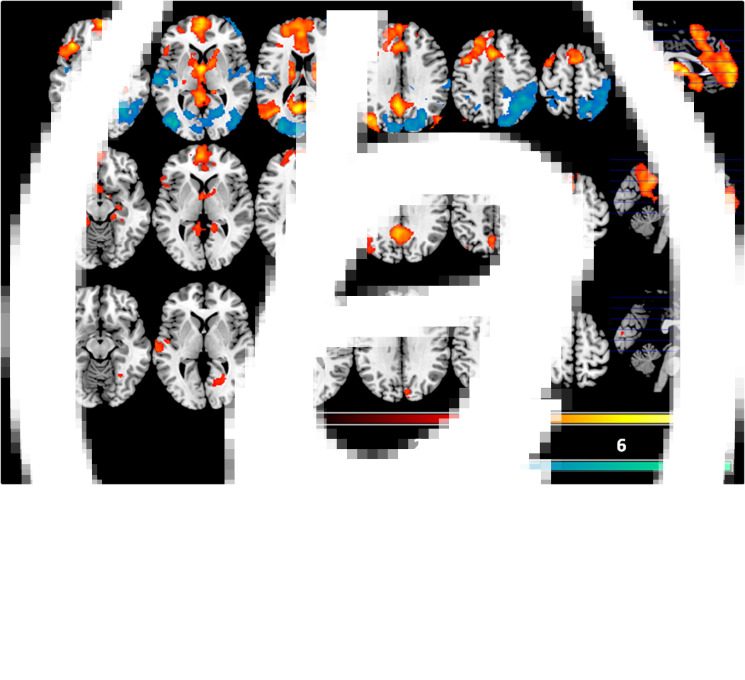
Areas of significant differences between the autobiographical memory-evoking and non-evoking cue conditions for the healthy subjects (a) and the schizophrenic patients (b). Warm colours represent autobiographical recall > non-memory-evoking cues, cold colours represent non-memory-evoking cues > autobiographical recall. Bottom row (c) shows areas of significant differences between the patients and controls in this contrast. Colour bars depict *Z* values. Images are displayed in neurological convention (right is right).

In this contrast the healthy controls showed a large cluster of greater activation to the memory-evoking cues than the non-memory-evoking cues in the medial frontal cortex extending to the orbitofrontal cortex and temporal poles bilaterally, as well as to the thalamus, the basal ganglia, the hippocampus and the parahippocampal cortex. This cluster also extended posteriorly to include a portion of the posterior cingulate cortex/precuneus and the calcarine cortex. Other clusters of activation included the left temporo-parietal junction (comprising the posterior portion of middle temporal cortex, the angular gyrus and the middle occipital cortex), the left middle temporal cortex, and the right angular gyrus (see Fig. 1, top panel, and online Supplementary Table S1).

The healthy controls also showed three clusters of increased activation to non-memory-evoking than to memory-evoking cues. These were in the occipital cortex bilaterally, the lateral parietal cortex bilaterally, more in the right hemisphere, the superior temporal cortex bilaterally and the right frontal polar cortex.

The schizophrenic patients (Fig. 1, middle panel) showed a broadly similar pattern of activation, although this appeared visually less extensive in the medial frontal cortex and subcortical regions. Unlike the controls, they showed no regions where there was relatively greater activation in response to non-memory-evoking cues.

Significant group differences (Fig. 1, bottom panel) were observed in four relatively small clusters: the right lingual gyrus [294 voxels, peak activation at BA 19, MNI (26, −54, −4), *z* score = 4.52, *p* = 0.003], the right cuneus [279 voxels, peak activation at BA 18, MNI (6, −90, 26), *z* score = 3.86, *p* = 0.004], the left middle temporal cortex [263 voxels, peak activation at BA 21, MNI (−68, −10, −2), *z* score = 4.48, *p* = 0.006] and the right angular gyrus [199 voxels, peak activation at BA 40, MNI (54, −52, 38), *z* score = 4.03, *p* = 0.02]. As can be seen from Fig. 1, these clusters of significant difference were all in regions where the healthy controls showed greater activation to non-memory-evoking cues than to memory-evoking cues. Box plots of mean activations within these four clusters are shown in online Supplementary Fig. S1. This confirmed that they all represented regions of relatively greater activation in the patients.

To investigate the possible influence of antipsychotic treatment on the above findings, the within-group analysis for the schizophrenic patients was repeated adding medication dose (in chlorpromazine equivalents) as a covariate. The findings in this group remained closely similar (see online Supplementary Material, Fig. S2A).

### fMRI findings: memory-evoking cues *v.* low level baseline

**Fig. 2.**
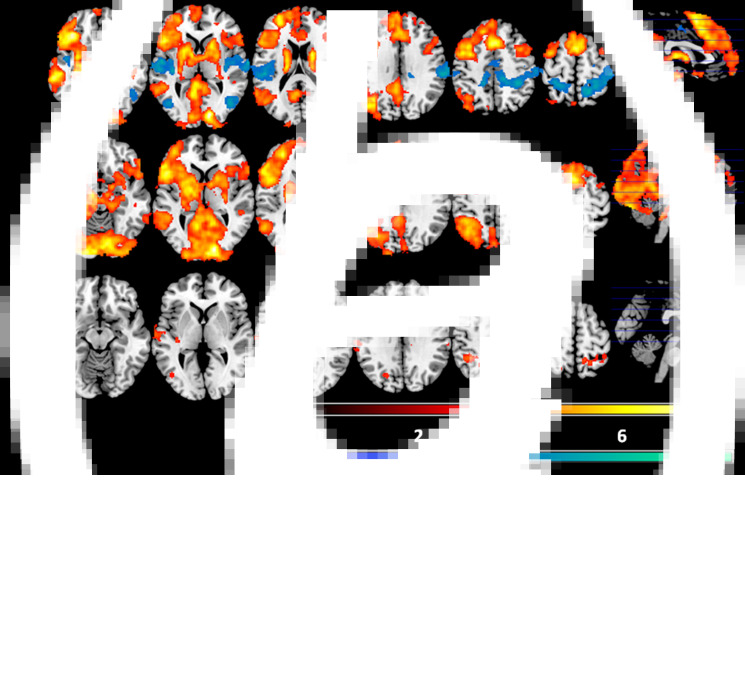
Activation map for the autobiographical memory-evoking cues *v*. fixation condition in the healthy subjects (a) and the schizophrenic patients (b). Warm colours represent autobiographical cues > baseline. Cold colours represent baseline > autobiographical cues. The third panel (c) shows areas of significant differences between the patients and the controls in this contrast. Colour bars depict *Z* values. Images are displayed in neurological convention (right is right).

Compared to cross fixation, the healthy controls showed a similar but more extensive pattern of activation than in the autobiographical memory-evoking *v.* non-evoking cue contrast. A large cluster encompassed the posterior cingulate cortex and precuneus, the left angular gyrus, the middle temporal cortex bilaterally, parts of the lateral prefrontal cortex and anterior insula bilaterally and the medial prefrontal cortex, and also extended to the occipital cortex, the hippocampus and parahippocampus, the thalamus, the basal ganglia and the cerebellum. A second cluster covered the posterior portion of the right middle temporal cortex and right angular gyrus. The third cluster of activation was located in the precuneus. The findings are shown in Fig. 2, top panel; further details are given in online Supplementary Table S2.

As can also be seen in Fig. 2, the healthy controls also showed clusters of de-activation compared to cross fixation. There were bilateral clusters in the superior temporal gyrus extending to the postcentral gyrus; the cluster on the right also extended to the superior parietal cortex and parts of the posterior cingulate cortex and precuneus. Two more bilateral clusters were seen in the inferior temporal cortex extending to the lateral occipital cortex. A fifth cluster was in the left superior parietal cortex.

The patients showed a broadly similar pattern of activation to the healthy controls, with large clusters in the medial prefrontal cortex and the posterior cingulate cortex/precuneus, hippocampus and parahippocampus, as well as in the lateral prefrontal cortex bilaterally, the left temporal and parietal cortex and parts of the occipital cortex bilaterally. However, they showed no clusters of de-activation (see Fig. 2, middle panel, and online Supplementary Table S2). As in the contrast between memory-evoking and non-evoking cues, repeating the within-group analysis for the schizophrenic patients adding medication dose (in chlorpromazine equivalents) as a covariate made little difference to the findings (see Supplementary Material, Fig. S2B).

There were no regions where the patients showed less activation compared to cross fixation than the controls (Fig. 2, bottom panel). However, the patients showed greater activation than the controls in seven clusters: the largest was in the right parietal cortex [1336 voxels, peak activation at BA 40, MNI (38, −44, 50), *z* score = 4.86, *p* < 0.001]; a roughly symmetrical but smaller cluster was in the left parietal cortex [563 voxels, peak activation at BA 40, MNI (−42, −46, 52), *z* score = 4.39, *p* < 0.001]; other clusters were in the postcentral gyrus [324 voxels, peak activation at BA 48, MNI (−64, −18, 22), *z* score = 4.08, *p* < 0.001]; the left middle temporal cortex [255 voxels, peak activation at BA 22, MNI (−62, −12, −2), *z* score = 5.01, *p* = 0.00329]; the left inferior temporal cortex [191 voxels, peak activation at BA 37, MNI (−48, −56, −8), *z* score = 4.58, *p* = 0.0145]; the left superior occipital cortex [163 voxels, peak activation at BA 19, MNI (−20, −74, 40), *z* score = 4.22, *p* = 0.029]; and the insula [148 voxels, peak activation at BA 48, MNI (−36, −20, 12), *z* score = 4.56, *p* = 0.0426].

Box plots of the mean activations within these seven clusters confirmed that in six cases they represented failure of de-activation in the schizophrenic patients (see Supplementary Fig. S3). The seventh cluster (in the superior occipital cortex) was in a region where the controls showed no significant activation or de-activation.

## Discussion

This study examined the brain functional correlates of autobiographical recall in schizophrenia, comparing it to two control tasks, viewing of non-memory evoking cues and cross fixation. Under both conditions the healthy controls showed activation within the territory of the default mode network, particularly in its two midline cortical regions. The patients did not differ significantly from the healthy controls in the degree of activation in these regions. However, they did show evidence of changes, which took the form mainly of failure of de-activation, in regions outside the default mode network.

The healthy controls in our study showed a pattern of autobiographical memory associated activations that was reasonably consistent with that found by Svoboda *et al*. (2006) in their meta-analysis of 24 studies. The most important difference was that, whereas Svoboda *et al*. (2006) found evidence of lateral frontal activation during task performance, in our study this was only seen under the memory cue evoking condition *v.* low level baseline (cross fixation), and not in the memory cue evoking *v.* non-evoking contrast. One possible explanation for this difference might be that the participants actively engaged in search (i.e. executive) strategies during both the active conditions, before either locating or failing to locate a relevant autobiographical memory. Some support for this explanation comes from a study of healthy subjects by Cabeza *et al*. (2004) which, as in our study, contrasted cues designed to both elicit and not elicit autobiographical memories (in this case, photographs of a university campus taken by the subject him-or herself or by others). Both conditions were found to activate the dorsolateral and ventrolateral prefrontal cortex compared to cross fixation, with no differences being found between the two active conditions.

Our findings in schizophrenic patients differ markedly from those of the only other study to date, that of Cuervo-Lombard *et al*. (2012). These authors found reduced activation in the patients in the medial frontal cortex and the precuneus, and also in the left lateral prefrontal cortex, the left medial temporal lobe and other areas, whereas we found no evidence of reduced activation in any region. However, there is an obvious potential explanation for this discrepancy: as noted in the introduction, Cuervo-Lombard *et al*.'s (2012) findings of reduced cortical activation in the schizophrenic patients were only obtained when a masked analysis using an uncorrected threshold was employed; whole brain corrected analysis revealed differences between the patients and the controls only in small clusters located in non-cortical regions.

Nevertheless, the schizophrenic patients in our study did show evidence of brain functional abnormality at the whole-brain corrected level. Clusters of significant difference were seen in both the memory-evoking *v.* non-memory evoking cues and in the memory-evoking cues *v.* low level contrasts, which took the form of relatively greater activation in both cases. As pointed out by Gusnard and Raichle (2001, see Methods), the interpretation of relative changes between two active tasks can be difficult, but the findings in the contrast between autobiographical memory-evoking cues and low-level baseline were clear: they represented failure of de-activation in the patients in six of the seven clusters of significant difference that emerged (the pattern in the seventh cluster, in the superior occipital cortex, was one of activation in a region where the controls showed neither activation nor de-activation). All these seven clusters were outside the regions usually considered to form part of the default mode network as identified by de-activations in studies using attention-demanding tasks (Buckner *et al*., 2008) or based on resting state connectivity (Yeo *et al*., 2011).

The obvious interpretation of this finding is that schizophrenic patients show failure of de-activation outside the default mode network during performance of a task that normally activates it. Clearly, however, such an interpretation depends on to what extent autobiographical recall can be considered to be normally associated with a pattern of non-default mode network de-activation. Unfortunately, this question is difficult to answer, as most studies of autobiographical recall in healthy subjects have not reported de-activations. Four early studies of autobiographical recall using PET noted both task related activations (i.e. recall > rest) and de-activations (i.e. rest > recall) (Andreasen *et al*., 1995; Fink *et al*., 1996; Gemar *et al*., 1996; Andreasen *et al*., 1999), but the sample sizes were mostly small (7–19 subjects) in these studies and the areas of de-activation varied widely. Only two fMRI studies appear to have reported de-activations. Ino *et al*. (2011) examined 21 healthy subjects and found greater activation under a ‘no thinking’ condition than during autobiographical recall in the temporal poles, orbitofrontal cortex, posterior insula, and portions of the bilateral middle/superior temporal, inferior parietal, and occipital cortex. De-activation was also observed in the mid-cingulate cortex and precuneus, extending into the superior parietal cortex. Bado *et al*. (2014) examined 18 healthy subjects and found greater activation during a ‘relax and stay awake’ condition than during recall of both emotional and neutral autobiographical memories in the subgenual anterior cingulate cortex, the ventral striatum and the hypothalamus/septal area, and additionally in the inferior parietal lobe bilaterally during recall of emotional memories. Collectively, these findings support the idea that autobiographical recall is associated with de-activations, and there are hints of an overlap with the findings from our own group of healthy controls.

The only other relevant data come from a study by DuPre *et al*. (2016) in which 31 healthy adults viewed emotional pictures and engaged in autobiographical recall, prospection or theory of mind reasoning based on their content. Data from this study are publicly available at NeuroVault (https://neurovault.org/collections/1866/). Contrasting self-generated thought (i.e. combining all three task conditions) to a baseline consisting of viewing a scrambled image followed by a button press, clusters of de-activation were seen in the parietal and temporal regions not dissimilar to those we observed in our study, as well as in the occipital cortex.

If replicated, the current study's finding of intact default mode activation but failure of de-activation outside the default mode network in schizophrenia would appear to have two main implications for the pathophysiology of the disorder. The first is that, rather than there being dysfunction of the default mode network specifically in schizophrenia, it may be that there is a more general problem with de-activation, which manifests itself in different regions (i.e. within or outside the default mode network) depending on the task used. Just such a proposal has recently been made by Allen *et al*. (2019), who argue that instead of schizophrenia being associated with ‘static’ dysfunctions in the prefrontal cortex and the default mode network, in reality the underlying brain dysfunction involves the dynamic balance between the ‘task positive’ networks (one of which is the fronto-parietal, executive or cognitive control network) and the ‘task negative’ or default mode network with which they are normally anticorrelated (Fox *et al*., 2005). Allen *et al*. (2019) go on to relate such a dysfunction to a change in the balance between the main excitatory and inhibitory transmitters in the brain, glutamate and GABA, although the evidence for dysfunction in the latter of these transmitter systems in schizophrenia is currently slender.

Second, the current study's findings bear on of the issue of how far default mode network dysfunction might underlie the cognitive impairment seen in schizophrenia. Anticevic *et al*. (2012) reviewed evidence on the relationship between default mode network activity and cognitive function in healthy subjects, noting that lower default mode network activity has been found to be associated with better performance across a number of cognitive tasks, and that higher levels of activity are correlated with lapses of attention. Based on this they suggested that the relevance of default mode network de-activation to cognition in schizophrenia warranted further investigation. So far, however, this investigation has been extremely limited. In what appears to be the only study to directly address this question, Ortiz-Gil *et al*. (2011) examined 18 cognitively impaired and 19 (relatively) cognitively preserved schizophrenic patients during performance of the n-back working memory task. They found that while the cognitively impaired patients showed hypoactivation compared to the cognitively preserved patients in the DLPFC and other regions, no differences in de-activation between the two groups were seen, even though the group of patients as a whole showed the expected failure of de-activation in the medial frontal cortex. The current study's finding of intact activation during autobiographical recall in schizophrenia adds a further, if currently not entirely clear, dimension to this debate.

In conclusion, the current study finds no evidence of altered default mode network function in schizophrenia during performance of a task which normally activates it, autobiographical recall. This finding needs to be viewed in the context of (a) conflicting findings in the only other study to use such a task in schizophrenia; and (b) the current lack of certainty that autobiographical recall is associated with de-activation outside the default mode network in healthy subjects. Further studies using not only autobiographical memory but perhaps also other tasks that activate the default mode network and, crucially, employing a low-level baseline in order to examine de-activations, would therefore be desirable. Some limitations need to be acknowledged. The sample sizes we employed were relatively small, and it is possible that activation differences between schizophrenic patients and controls may have emerged if these were larger. The schizophrenic patients were taking antipsychotic medication, and this potential confounding factor is not easy to fully address in a study making comparisons with healthy subjects. We did not measure autobiographical memory performance in the patients and so there is an unanswered question about whether and to what extent task related activations (and de-activations) were influenced by poor task performance.

## Supplementary material

For supplementary material accompanying this paper visit https://doi.org/10.1017/S0033291719003052. click here to view supplementary material
