# Autobiographical memory and default mode network function in schizophrenia: an fMRI study—CORRIGENDUM

**DOI:** 10.1017/S0033291720001361

**Published:** 2021-01

**Authors:** Marta Martin-Subero, Paola Fuentes-Claramonte, Pilar Salgado-Pineda, Josep Salavert, Antoni Arevalo, Clara Bosque, Carmen Sarri, Amalia Guerrero-Pedraza, Aniol Santo-Angles, Antoni Capdevila, Salvador Sarró, Raymond Salvador, Peter J. McKenna, Edith Pomarol-Clotet

In the above article, affiliation 3 was published with the department missing and should be ‘Department of Psychiatry and Forensic Medicine. Universitat Autònoma de Barcelona, Barcelona, Spain’

This has now been corrected in the online version of the article. The authors apologise for this error.
