# Supplementary material for: Autobiographical memory and default mode network function in schizophrenia: an fMRI study
Source: Psychol Med. 2019 Nov 4;51(1):121–8. doi: 10.1017/S0033291719003052 (PMC7856411; doi:10.1017/S0033291719003052)
Supplement: Supplementary file 1 [file S0033291719003052sup001.docx]

**Supplementary Material**

**Table S1.** Regions of activation and de-activation in the autobiographical recall vs non-memory-evoking conditionn.

|  | **MNI coordinates** | | |  |  |  |
| --- | --- | --- | --- | --- | --- | --- |
| **Region/Contrast** | **x** | **y** | **z** | **Z-value** | ***k*** | ***p*** |
| *Controls*  *recall > non-recall* |  |  |  |  |  |  |
| Thalamus | -2 | -4 | 4 | 7.09 | 17834 | *p* < 0.001 |
| Precuneus | 8 | -56 | 22 | 6.96 |  |  |
| Cerebellum | 36 | -62 | -28 | 6.69 |  |  |
| Calcarine cortex | 4 | -56 | -16 | 6.59 |  |  |
| Medial prefrontal cortex | -2 | 62 | 6 | 6.32 | 10515 | *p* < 0.001 |
| Supplementary motor area | -2 | 18 | 48 | 5.67 |  |  |
| Anterior cingulate cortex | -4 | 46 | 16 | 5.55 |  |  |
| Middle occipital cortex | -46 | -74 | 34 | 6.25 | 1671 | *p* < 0.001 |
| Middle temporal cortex | -50 | -70 | 20 | 5.54 |  |  |
| Middle temporal cortex | -62 | -8 | -18 | 4.47 | 444 | *p* < 0.001 |
| Angular gyrus | 50 | -74 | 32 | 4.51 | 201 | *p* = 0.020 |
| *Controls*  *Non-recall > recall* |  |  |  |  |  |  |
| Middle occipital cortex | -42 | -82 | 8 | 6.27 | 19270 | *p* < 0.001 |
| Superior occipital cortex | 28 | -74 | 38 | 6.24 |  |  |
| Superior parietal cortex | 20 | -56 | 60 | 6.03 |  |  |
| Superior temporal cortex | -50 | -10 | 0 | 6.06 | 1929 | *p* < 0.001 |
| Middle temporal cortex | -60 | -20 | 2 | 5.19 |  |  |
| Frontal pole | 30 | 68 | 10 | 5.66 | 692 | *p* < 0.001 |
| *Patients*  *recall > non-recall* |  |  |  |  |  |  |
| Medial prefrontal cortex | -4 | 56 | 2 | 5.39 | 3372 | *p* < 0.001 |
| Anterior cingulate cortex | -4 | 28 | 32 | 4.16 |  |  |
| Posterior cingulate cortex | -4 | -48 | 34 | 5.72 | 3248 | *p* < 0.001 |
| Precuneus | -6 | -54 | 24 | 5.66 |  |  |
| Parahippocampal gyrus | -12 | -2 | -20 | 4.40 | 1032 | *p* < 0.001 |
|  | 24 | -20 | -20 | 5.18 | 878 | *p* < 0.001 |
| Superior frontal cortex | -16 | 36 | 54 | 4.87 | 803 | *p* < 0.001 |
| Angular gyrus | -42 | -62 | 28 | 3.97 | 489 | *p* < 0.001 |
| Middle temporal cortex | -62 | -22 | -16 | 4.46 | 435 | *p* < 0.001 |
|  | 62 | -6 | -22 | 4.88 | 226 | *p* = 0.012 |
| Temporal pole | -58 | 10 | -12 | 3.90 | 251 | *p* = 0.007 |

**Table S2.** Regions of activation and de-activation in the autobiographical recall condition compared to fixation.

|  | **MNI coordinates** | | |  |  |  |
| --- | --- | --- | --- | --- | --- | --- |
| **Region/Contrast** | **x** | **y** | **z** | **Z-value** | ***k*** | ***p*** |
| *Controls: activation* |  |  |  |  |  |  |
| Calcarine cortex | 16 | -86 | 2 | 8.14 | 58713 | *p* < 0.001 |
| Cerebellum | 36 | -62 | -28 | 8.09 |  |  |
| Inferior frontal cortex | -38 | 30 | -10 | 7.87 |  |  |
| Middle temporal cortex | -64 | -34 | -10 | 7.14 |  |  |
| Middle temporal cortex | 58 | -58 | 16 | 5.03 | 598 | *p* < 0.001 |
| Precuneus | -6 | -74 | 56 | 3.97 | 144 | *p* = 0.047 |
| *Controls: de-activation* |  |  |  |  |  |  |
| Postcentral gyrus | 22 | -42 | 76 | 6.17 | 7728 | *p* < 0.001 |
| Superior temporal gyrus | 52 | -30 | 14 | 5.96 |  |  |
| Mid-cingulate cortex | 12 | -30 | 42 | 5.95 |  |  |
| Superior parietal cortex | 20 | -50 | 64 | 5.78 |  |  |
| Heschl gyrus | -38 | -20 | 12 | 6.95 | 3285 | *p* < 0.001 |
| Superior temporal gyrus | -54 | -8 | 0 | 6.05 |  |  |
| Supramarginal gyrus | -64 | -26 | 30 | 5.49 |  |  |
| Rolandic operculum | -50 | -26 | 16 | 5.33 |  |  |
| Superior parietal cortex | -22 | -44 | 66 | 5.30 | 1079 | *p* < 0.001 |
| Inferior temporal cortex | 50 | -68 | -4 | 5.48 | 859 | *p* < 0.001 |
| Middle occipital cortex | -46 | -72 | 4 | 4.66 | 242 | *p* = 0.004 |
| *Patients: activation* |  |  |  |  |  |  |
| Calcarine cortex | 14 | -90 | 2 | 8.16 | 69677 | *p* < 0.001 |
| Cerebellum | 10 | -82 | -16 | 7.46 |  |  |
| Lingual gyrus | -18 | -94 | -12 | 7.05 |  |  |
| Inferior frontal cortex | -54 | 16 | 0 | 7.01 |  |  |
| Angular gyrus | 36 | -58 | 50 | 4.22 | 240 | *p* = 0.005 |
| Middle temporal cortex | 52 | -34 | -4 | 4.39 | 142 | *p* = 0.049 |


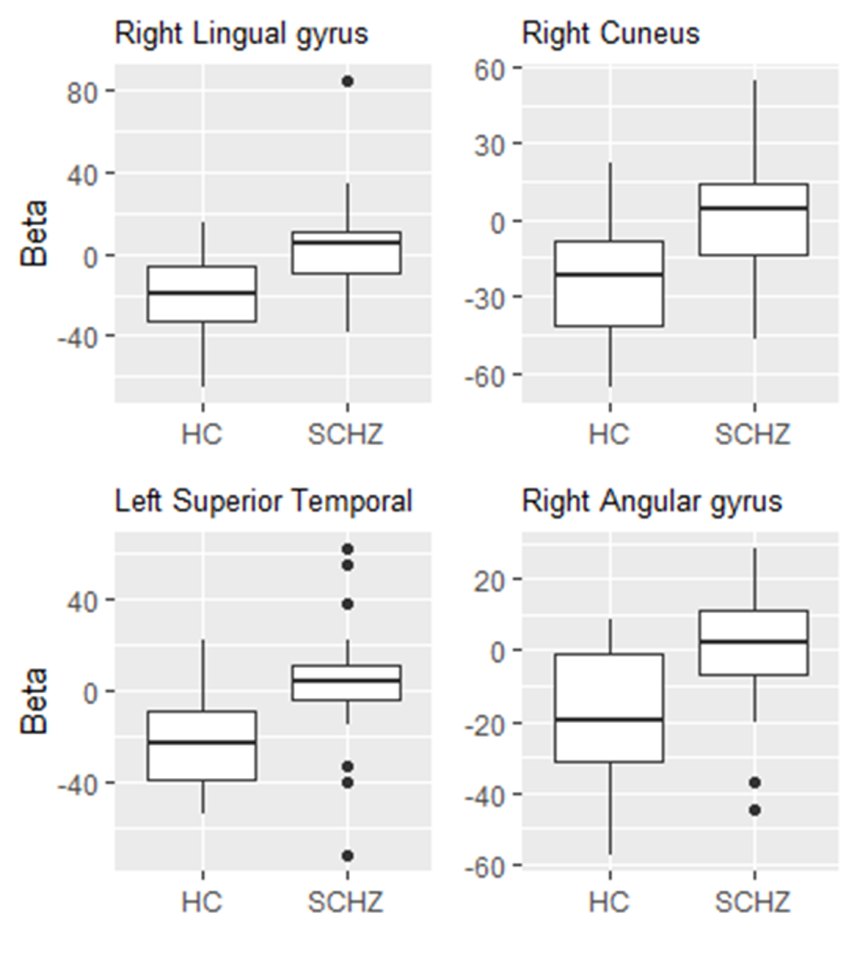


**Figure S1.** Boxplots showing activation levels in patients and controls for the four clusters of differences between groups in the autobiographical memory evoking vs non-evoking cues contrast. Y-axis depicts parameter estimates (beta values).

**
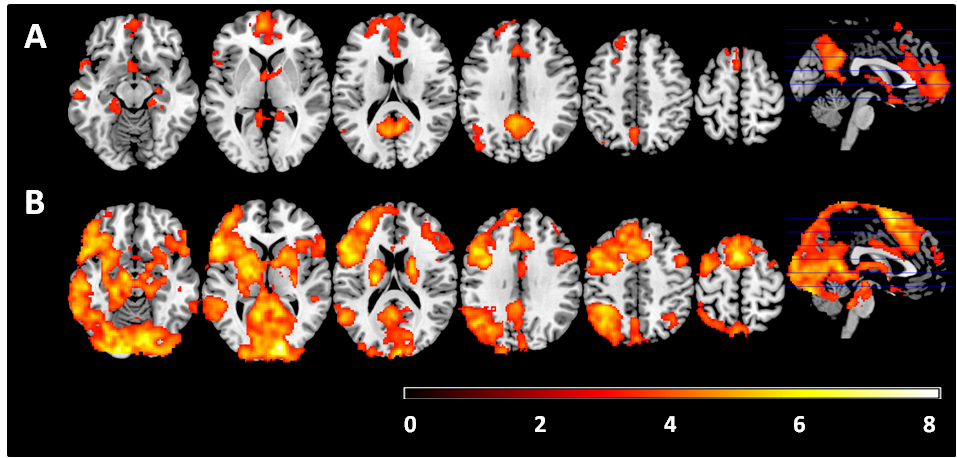
**

**Figure S2.** Mean activation maps for the patient group, controlling for antipsychotic dose (in chlorpromazine equivalents): (A) Areas of significant differences between the autobiographical memory-evoking and non-evoking cue conditions. (B) Areas of significant differences between the autobiographical memory-evoking cues and fixation conditions. Colour bars depict Z values. Images are displayed in neurological convention (right is right).

**
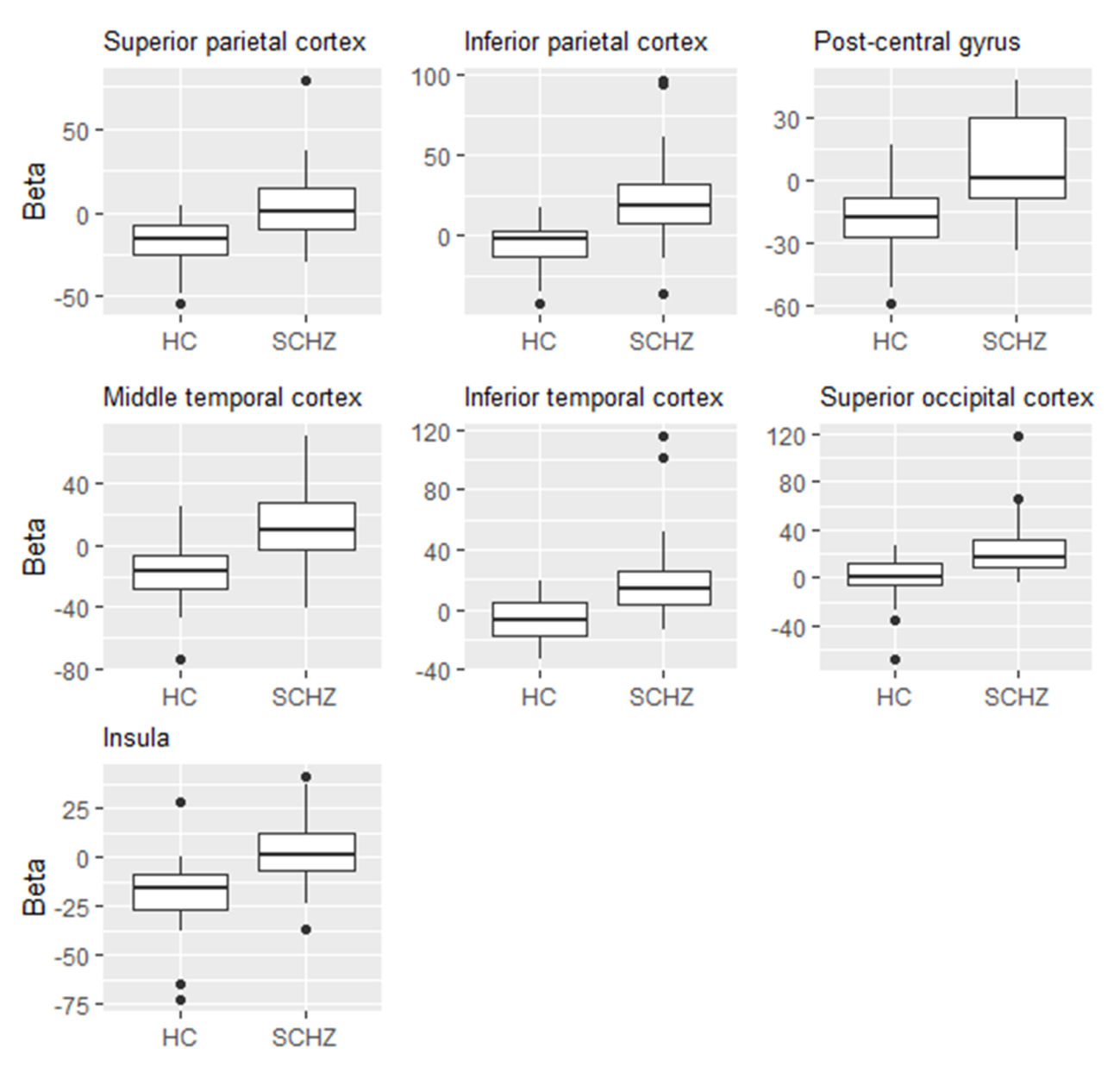
**

**Figure S3.** Boxplots showing activation levels in patients and controls for the seven clusters of differences between groups in the autobiographical memory evoking vs fixation contrast. Y-axis depicts parameter estimates (beta values).
